# Outbreak of *Serratia marcescens* in the Neonatal Intensive Care Unit of a Tertiary Care Hospital in Mexico

**DOI:** 10.1155/2023/3281910

**Published:** 2023-09-21

**Authors:** Martha Guel-Gomez, Uriel A. Angulo-Zamudio, Nidia Leon-Sicairos, Hector Flores-Villaseñor, Edna Mendívil-Zavala, Amparo Plata-Guzmán, Jesus J. Martinez-Garcia, Jorge Angulo-Rocha, Rosangela Ochoa-Espinoza, Paola Crespo-Palazuelos, Jesús Bracamontes-Murillo, Angel León-Ramírez, Juan C. Rodriguez-Ceceña, Adrian Canizalez-Roman

**Affiliations:** ^1^The Women's Hospital, Secretariat of Health, Culiacan Sinaloa 80020, Mexico; ^2^School of Medicine, Autonomous University of Sinaloa, Culiacan Sinaloa 80019, Mexico; ^3^Pediatric Hospital of Sinaloa, Culiacan Sinaloa 80200, Mexico; ^4^The Sinaloa State Public Health Laboratory, Secretariat of Health, Culiacan Sinaloa 80020, Mexico

## Abstract

**Introduction:**

We describe an outbreak of *Serratia marcescens* (*S. marcescens*) infection in the neonatal intensive care unit at Women's Hospital in Sinaloa, Mexico.

**Methods:**

In April 2021, an outbreak of *S. marcescens* infection was identified. A case was identified as any patient who tested positive for *S. marcescens* and showed signs of an infectious process.

**Results:**

*S. marcescens* was isolated from the blood cultures of 15 neonates with clinical signs of neonatal sepsis. Statistical analysis showed that all neonates had an invasive medical device. The problem was controlled after hospital hygiene and sanitation measures were strengthened.

**Conclusion:**

The study provides evidence of an outbreak of nosocomial bacteremia due to the cross-transmission of *S. marcescens.* The findings highlight the need for hospitals to implement strict hygiene measures, especially regarding hand washing, to prevent future outbreaks.

## 1. Introduction

*Serratia marcescens* (*S. marcescens*) is an aerobic, Gram-negative bacillus of the Enterobacteriaceae family with multiple virulence factors, including several enzymes and the ability to form biofilms; this bacterium is found in the normal microbiota of the human gut. Epidemic outbreaks of *S. marcescens* have been reported with mechanical ventilators and disinfectants such as chlorhexidine [1, 2], soaps, stethoscope [3], incubator [4] parenteral solutions [5, 6], infant shampoo [7], and hands as possible sources of transmission. Based on the abovementioned, this pathogen has been classified as an opportunistic pathogen in hospitalized patients causing nosocomial outbreaks [8].

Several risk factors have been identified in outbreaks of *S. marcescens*, such as prematurity [9], in severely immunocompromised or critically ill patients of all ages [10], especially in intensive care units (ICUs) and in neonatal intensive care units (NICUs) [11], causing healthcare-associated infections (HAIs) due to this germ [12, 13]. In the United States, Serratia species are responsible for 1.4% of nosocomial bacteremias. In Mexico, it is estimated that 450,000 cases of nosocomial infections occur annually, causing 32 deaths per 100,000 inhabitants per year (whose annual cost of care is nearly 1.5 billion) [14].

*S. marcescens* can cause asymptomatic or symptomatic infections with a wide range of clinical manifestations [15]. Among the symptoms are keratitis, conjunctivitis, urinary tract infections, pneumonia, surgical wound infections, sepsis, meningitis, and bloodstream infections [11, 16, 17]. It is the third most common pathogen causing epidemic outbreaks [18].

In recent decades, this microorganism has become more critical as it is responsible for outbreaks that are difficult to control with multidrug-resistant strains, especially in NICUs [11, 19]. The high survival capacity of the microorganism in the hands of healthcare workers [20], prolonged stay, use of invasive medical devices, and previous administration of antibiotics during pregnancy are described as risk factors for its acquisition.

In Mexico, antimicrobial resistance [21] and virulence profiles [22] have been evaluated in *S. marcescens* isolates from different regions of the country. Similarly, outbreaks of nosocomial bacteremia and colonization by *S. marcescens* have been reported in an intensive care unit (ICU) [23] and the neonatal intensive care unit (NICU) [24]. In Sinaloa, no evidence addresses this type of problem. Hence, the description of the outbreak of *Serratia marcescens* is of great relevance to seeking alternatives that help prevent and manage infections caused by this type of pathogens, especially in the intrahospital setting.

The purpose of presenting a case series of an epidemic outbreak caused by *S. marcescens* with its clinical and epidemiological characteristics is to improve patient-centered care and avoid new healthcare-associated infections or early and timely detection to prevent or reduce morbidity and mortality.

## 2. Materials and Methods

An outbreak of 15 cases of neonatal *S. marcescens* infection from April to May 2021 and records before the 2020 outbreak were analyzed. The cases were identified in the neonatal unit of the Women's Hospital in Sinaloa, located in northwestern Mexico. The hospital has a capacity of 32 census beds and an average birth rate of 500 newborns per month. All newborns with a positive blood culture for *S. marcescens*, with or without clinical symptoms, were included as confirmed cases during the abovementioned period. An outbreak was defined as two or more cases of healthcare-associated infections (HAIs) acquired by patients, healthcare workers, and/or visitors to the hospital unit [25, 26]. *S. marcescens* was isolated from blood cultures processed by the Women's Hospital staff.

In addition, a follow-up was performed until May 2022, during which the medical records of all suspected cases of infection were analyzed. In addition, possible sources of *S. marcescens* contamination were identified, and environmental cultures were collected from neonatal areas, equipment, supplies, water, soap, antiseptics, milk, and staff hands. The present study was approved by the Ethics Committee of the Women's Hospital (no. 202206-12).

## 3. Results

In 2020 (before the outbreak), there were 6,548 births at the Women's Hospital of Sinaloa, with no reported cases of *S. marcescens*. In 2021, 4,698 newborns were born, of which 15 had nosocomial *S. marcescens* infection, with an incidence of 0.0031 per 1000 newborns. Out of the fifteen neonates who were infected with *S. marcescens*, nine were taken to the NICU, five were taken to intermediate care, and one was placed in the ward with their mother. It is important to note that all the neonates were from the neonatal transitional zone.

**Figure 1:**
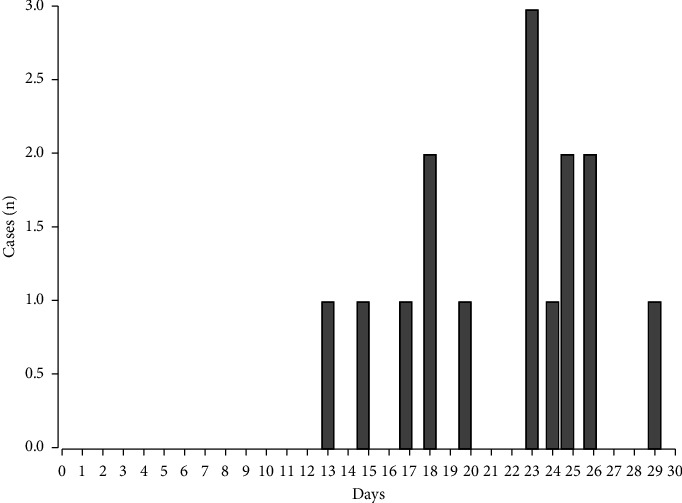
Epidemiologic curve of the *S. marcescens* outbreak in the neonatal intensive care unit of the Women's Hospital in Sinaloa, Mexico.

The first neonate (primary index case) was born on April 10, 2021, and was admitted to the NICU with a diagnosis of transient tachypnea of the newborn (TTRN). The neonate displayed clinical manifestations within 4 hours of life, which included symptoms such as vomiting, tachypnea, and an abrupt onset of shock. The neonate's culture was positive for *S. marcescens* 48 hours after birth, and cases began to appear after that, which was considered an epidemic outbreak.  Figure 1 shows the epidemiologic course of the affected neonates.

The 15 infected neonates were born to Mexican mothers, of whom 20% (*n* = 3/15) were adolescents and 80% (*n* = 12/15) were adults, with ages ranging from 15 to 39 years and a mean age of 24.6 years. All the mothers had at least one risk factor for neonatal sepsis, the most common being maternal infection (27% (*n* = 4/15)), hypertension (27% (*n* = 4/15)), and diabetes (20% (*n* = 3/15)). The mean number of antenatal visits was 5.8.

The distribution of the neonatal delivery route was as follows: 47% (*n* = 7/15) vaginal and 53.3% (*n* = 8/15) abdominal. Forty-seven percent (*n* = 7/15) of the neonates were preterm (equal to or less than 36 weeks' gestation (WG)) and 53% (*n* = 8/15) were term (37 to 42 WG), with a minimum of 32 WG, a maximum of 40, and a mean of 36.9 WG. 13% (*n* = 2/15) weighed less than 2500 g, and 87% (*n* = 13/15) weighed more than 2500 g with a minimum of 1500 g, a maximum of 3500 g, and a mean of 2912 g. Of the 15 neonates, 93% (*n* = 14/15) were born with adequate weight for their gestational age and 7% (*n* = 1/15) with high weight for gestational age. Of the neonates, 73.3% (*n* = 11/15) were identified as male and 26.6% (*n* = 4/15) as female. The mean length of hospital stays before *S. marcescens*isolation ranged from 4 to 30 hours after birth. The mean length of hospital stay was 19 days (minimum 5 and maximum 40) (Table 1).

93% (*n* = 14/15) of the neonates had an invasive medical device before infection, such as vascular access; 66% (*n* = 10/15) had an umbilical catheter, 26.6% (*n* = 4/15) had a peripheral catheter, only one neonate had no prior vascular access or another type of device, and 20% (*n* = 3/15) received previous antibiotic treatment, as shown in Table 1. Regarding feeding, 93% (*n* = 14/15) received mixed feeding (combination of breast milk and formula). Twenty percent (*n* = 3/15) of the neonates received prior antibiotic treatment.

The type of infection observed was neonatal sepsis in 93% (*n* = 14/15), with respiratory changes predominating in 66% (*n* = 10/15), jaundice was present in 80% (*n* = 12/15) of the neonates, 47% (*n* = 7/15) had gastrointestinal manifestations, thrombocytopenia was present in 80% (*n* = 12/15), fever and dysthermia were present in 73.3% (*n* = 11/15), and earthy skin with color changes was observed mainly in 60% (*n* = 9/15), as shown in Table 1. Regarding biochemical parameters, procalcitonin was elevated in 86.6% (*n* = 13/15) of the neonates and C-reactive protein (CRP) in 80% (*n* = 12/15) (Supplementary Table 1). Only the index case died on the fifth day of life and hospitalization, resulting in a case fatality rate of 7%. The *S. marcescens* strain was susceptible to most antimicrobials, and the neonates received mainly ampicillin, amikacin, cefotaxime, and some also meropenem, among others.

In addition, cultures were obtained from potential fomites (medical equipment, areas, supplies, and personnel) (Supplementary Table 2). The follow-up of the outbreak continued for one year, and the results of cultures collected from neonates admitted to the NICU during this time were monitored, with both preoutbreak and outbreak cultures being negative for *S. marcescens.*

## 4. Discussion

Therefore, *S. marcescens* is widely distributed in the environment and is particularly associated with hospital-acquired infections and hospital-associated outbreaks. More than 200 hospital outbreaks caused by *S. marcescens* have been reported in the medical literature since 1950 [27]. Outbreaks have affected pediatric [19] and adult populations, immunocompromised hosts, and intensive care units (ICUs) [28]. In this study, an outbreak of *S. marcescens* was identified in 15 neonates at the Women's Hospital of Sinaloa, only one of whom died.

According to Canadian studies, the incidence of Serratia infections is estimated at 10.8 per 100,000 persons per year, with a hospital incidence rate of 0.4 per 1,000 hospitalized patients [13]. On the other hand, a multicenter study of premature infants in 774 hospitals in the United States showed an increased incidence of invasive Serratia, corresponding to approximately 2.3 Serratia infections per 1,000 premature infants [29]. Both studies showed a higher incidence than that found in our work. Most infants were transferred on average at about 20 hours of age, which may seem like a long time; however, the specificity of sepsis symptoms in the first 24 hours has low specificity, resulting in long transfer times to the ICU [30]. On the other hand, time to care for neonates with sepsis is very important; it has been estimated that the likelihood of neonatal mortality can increase by up to 1.5% for every hour of delay in transferring these patients to the ICU [31]. More effective tools are needed to identify neonatal sepsis as early as possible and provide the best treatment.

Regarding Mexico, a study conducted in a NICU at the Children's Hospital of Mexico between May 2005 and July 2006 reported an outbreak of *S. marcescens*. In this outbreak, only one case of *S. marcescens* was identified in neonates between May 2005 and May 2006, corresponding to 0.08 cases/14 admissions/month; after placing the first case, these increased to 7/14 admissions/month. No cases were identified in the month of follow-up after the outbreak [32]. The data from this outbreak are consistent with those reported in our study since the outbreak was stopped thanks to control measures.

In this work, 80% of the cases corresponded to males and 20% to females, which is similar to what has been reported in another outbreak [2], but at the same time, different from what has been reported by other researchers [15, 24], probably because when the outbreak occurs, it can affect both males and females equally. On the other hand, most of the cases corresponded to neonates born at term with a birth weight of more than 2,500 grams, which contrasts with what has been reported by other authors who identify low birth weight and prematurity as risk factors for morbidity and mortality due to this type of pathogen [19].

Regarding sepsis, the predominant manifestations in this study were generalized changes, jaundice, respiratory distress, and thrombocytopenia. These data are consistent with those reported by Voeltz et al., who, in a systematic review of 27 studies of *S. marcescens* outbreaks in neonatal and pediatric intensive care units, identified symptoms similar to those in our study [19].

In addition, most of the neonates in this study were exposed to a device placed in the airway, which is a significant risk factor for *S. marcescens* infection, which is consistent with the literature that medical devices are a risk factor for contamination and transmission of these pathogens [5]. However, despite multiple cultures in the environment and on devices, supplies, equipment, and materials, *S. marcescens* was not detected in the cultures. In addition, only 20% had received prior antibiotic treatment, which is in contrast to what other studies have reported, as this is a risk factor for the development of nosocomial infections due to early exposure to such drugs with consequent high antimicrobial resistance [33].

The evidence from our study shows that the outbreak had a horizontal transmission mechanism as the same bacterial strain was isolated in all cases. The index patient rapidly evolved with predominantly respiratory and gastrointestinal symptoms and severe sepsis. Contrary to what has been reported in the literature, where prolonged hospital stay is a significant risk factor, in this case series, the hospital stay before the onset of infection was very short, less than 30 hours.

It is now recognized that nosocomial outbreaks can result from poor hygienic practices and contamination of materials within a hospital, facilitating infection transmission and affecting many patients. In the present report, an outbreak of neonatal *S. marcescens* infection was identified in 15 cases, one of which died (case fatality rate of 7%). According to different reports, the case fatality rate of *S. marcescens* infection varies widely, ranging from 8% to 69% [34–37], although the trend is high in most reports. In this study, however, a low mortality rate was reported, possibly because of rapid detection and because *S. marcescens* was susceptible to a wide range of antibiotics, which helped to eradicate infections.

This study had the limitation that bacterial genotyping studies were not performed because blood culture samples were discarded. In addition, the lack of fecal analysis of the exposed neonates and the index patient, who had early gastrointestinal and respiratory data until the development of severe sepsis, might have revealed the origin of the infection via the gastrointestinal route and its subsequent dissemination via the hematogenous route. Although *S. marcescens* tends to colonize the human gastrointestinal tract, preventive measures should be promoted and implemented, with emphasis on those involving direct contact with the patient, by performing hand disinfection activities, using new gloves, clean medical equipment, and exclusive clothing such as gowns for the care of each neonate.

## 5. Conclusion

The epidemiologic analysis of this study provides evidence of an outbreak of nosocomial bacteremia that started in the transitional area and spread from there to different areas (NICU, intermediate care, and wards), probably caused by cross-transmission of *S. marcescens* by personnel or an invasive medical device, since the source of transmission could not be identified. On the other hand, it is essential to emphasize that different intervention strategies to contain outbreaks should focus on basic daily activities such as proper hand washing, use of clean medical equipment, and disinfection of other areas within a hospital to significantly reduce prolonged stays, morbidity, and mortality from these diseases, as well as the costs generated by neonatal sepsis.

## Figures and Tables

**Table 1:** Clinical characteristics of the *S. marcescens* outbreak cases in the neonatal intensive care unit of the Women's Hospital in Sinaloa, Mexico.

Nos.	Gender	Admission age (hours)	Admitting diagnosis	Pregnancy status (w)	Admission weight (g)	Hospital days before *S. marcescens* isolation	Procedures before the symptoms	Previous antibiotics therapy	Symptoms	Antibiotics therapy	Final diagnosis	Hospital days until discharge	Outcome
1	M	4	TTN	Term (39)	2870	2	CPAP, nasal tips, peripheral catheter, parenteral solutions	Not	Respiratory distress, vomiting, abdominal distension, jaundice, hepatosplenomegaly, fever, pallor, skin color changes, thrombocytopenia, and ventricular hemorrhage	Yes	Sepsis respiratory	5	Death

2	F	24	TTN/SDR	Term (40)	2900	1	CPAP, intubation, mechanical ventilation, umbilical catheter	Not	Respiratory distress, jaundice, fever, thrombocytopenia	Yes	Sepsis respiratory	18	Health improvement

3	M	11	TTN/ARDS/respiratory depression	Preterm (36)	3250	1	NIPPV, intubation, umbilical catheter, mechanical ventilation, parenteral solutions	Not	Respiratory distress, abdominal distension, jaundice, hepatosplenomegaly, fever, pallor, skin color changes, hypoactivity	Yes	Sepsis respiratory	21	Health improvement

4	F	24	APGAR low score/neonatal asphyxia	Term (39)	3200	1	NIPPV, peripheral catheter, parenteral solutions	Not	Respiratory distress, jaundice, fever, pallor, thrombocytopenia	Yes	Sepsis respiratory	20	Health improvement

5	M	6	Sepsis risk by poor prenatal control	Term (39)	3150	2	Peripheral catheter, parenteral solutions	Yes	Respiratory distress, vomiting, abdominal distension, fever, thrombocytopenia	Yes	Sepsis respiratory	21	Health improvement

6	M	24	TTRN/ARDS	Preterm (32)	1600	1	CPAP, intubation, umbilical catheter, and parenteral solutions	Not	Respiratory distress, jaundice, fever, pallor, thrombocytopenia, and lung hemorrhage	Yes	Sepsis respiratory	30	Health improvement
7	M	27	TTRN/ARDS	Term (39)	2930	3	CPAP, nasal tips, umbilical catheter, and parenteral solutions	Not	Respiratory distress, abdominal distension, skin color change, hypoactivity, thrombocytopenia, digestive hemorrhage	Yes	Sepsis respiratory	14	Health improvement

8	M	24	TTN/ARDS/sepsis risk by maternal cervicovaginitis	Preterm (36)	2470	4	NIPPV, CPAP, intubation, umbilical catheter, parenteral solution, orogastric tube	Yes	Respiratory distress, jaundice, fever, pallor, skin color change, hypoactivity, digestive-ventricular hemorrhages	Yes	Sepsis respiratory	40	Transferred^*∗*^ to another hospital

9	M	18	TTN/ARDS/neonatal asphyxia/perinatal asphyxia/MAS	Term (39)	3400	8	NIPPV, intubation, umbilical catheter, and parenteral solutions	Not	Jaundice, fever, skin color change, thrombocytopenia	Yes	Sepsis respiratory	27	Health improvement

10	F	6	TTN/ARDS	Preterm (35)	2700	1	NIPPV, CPAP, umbilical catheter, and parenteral solutions	Not	Respiratory distress, abdominal distension, jaundice, hepatosplenomegaly, fever, skin color change, hypoactivity, thrombocytopenia, digestive hemorrhage	Yes	Sepsis respiratory	15	Health improvement

11	M	36	TTN/ARDS	Term (37)	2620	5	CPAP, nasal tips, umbilical catheter, peripheral catheter, and parenteral solutions	Not	Vomit, jaundice, hepatosplenomegaly, hypoactivity, thrombocytopenia	Yes	Sepsis respiratory	16	Health improvement
12	M	48	TTN/ARDS	Term (38)	3320	3	CPAP, nasal tips, umbilical catheter, and parenteral solutions	Not	Jaundice, fever, pallor, skin color change, thrombocytopenia	Yes	Sepsis respiratory	15	Health improvement

13	M	20	TTN/ARDS	Preterm (36)	3500	1	CPAP, umbilical catheter, and parenteral solutions	Yes	Respiratory distress, abdominal distension, jaundice, hepatosplenomegaly, fever, pallor, skin color change, hypoactivity, hemorrhages	Yes	Sepsis respiratory	17	Health improvement

14	M	11	TTN/ARDS/neonatal asphyxia	Preterm (36)	2910	1	NIPPV, CPAP, intubation, mechanical ventilation, umbilical catheter, and parenteral solutions	Not	Skin color changes, hypoactivity, and thrombocytopenia	Yes	Sepsis respiratory	13	Health improvement

15	F	31	Neonatal jaundice/early-onset neonatal sepsis	Preterm (35)	2930	1	No procedures	Not	No symptoms	Yes	Colonization	11	Health improvement

Nos.: numbers, TTN: transient tachypnea of the newborn, ARDS: acute respiratory distress syndrome, MAS: meconium aspiration syndrome, w: weeks, NIPPV: intermittent positive pressure ventilation, and CPAP: continuous positive airway pressure. ^*∗*^: the patient was transferred for requiring procedures that the hospital did not have, and the patient left the hospital infection-free.

## Data Availability

The datasets generated and/or analyzed during the current study are available from the corresponding author upon reasonable request.

## Supplementary Materials

Supplementary Materials Supplementary Table 1: laboratory parameters of neonates infected with *S. marcescens*. Laboratory parameters such as leukocytes, platelets, procalcitonin, and C-reactive protein (CRP) in newborn infants who have been infected with the pathogen. Supplementary Table 2: screening for *S. marcescens* in samples collected from various areas of the Women's Hospital. Samples for bacterial culture were collected from floors and walls, air conditioners, oxygen and air intakes, furniture, medical equipment (cribs, incubators, and ventilators), water, milk, supplies, catheters, soap, computer equipment, and staff hands.
