# Supplementary material for: Outbreak of Serratia marcescens in the Neonatal Intensive Care Unit of a Tertiary Care Hospital in Mexico
Source: Adv Med. 2023 Sep 21;2023:3281910. doi: 10.1155/2023/3281910 (PMC10539092; doi:10.1155/2023/3281910)
Supplement: Supplementary Materials — Supplementary Table 1: laboratory parameters of neonates infected with S. marcescens. Laboratory parameters such as leukocytes, platelets, procalcitonin, and C-reactive protein (CRP) in newborn infants who have been infected with the pathogen. Supplementary Table 2: screening for S. marcescens in samples collected from various areas of the Women's Hospital. Samples for bacterial culture were collected from floors and walls, air conditioners, oxygen and air intakes, furniture, medical equipment (cribs, incubators, and ventilators), water, milk, supplies, catheters, soap, computer equipment, and staff hands. [file 3281910.f1.zip › Supplementary Table 1 (1).docx]

| **Supplementary Table 1**. Laboratory parameters of neonates infected with *S. marcescens.* | | | | |  |
| --- | --- | --- | --- | --- | --- |
| **N°** | **leukocytes (mm3)** | **Platelets (mm3)** | **Procalcitonin (ng/ml)** | **CRP (mg/dL)** |  |
|  |  |  |  |  |  |
| 1 | 19830 | 14 000 | 121.62 | - |  |
| 2 | 22229 | 167000 | - | 3.97 |  |
| 3 | 19580 | 205000 | 93.19 | 20.91 |  |
| 4 | 27000 | 50000 | 23.19 | 9.58 |  |
| 5 | 16070 | 14000 | 14.92 | 31.19 |  |
| 6 | 22650 | 54000 | 299.36 | 13.69 |  |
| 7 | 12970 | 6000 | 162 | 33.06 |  |
| 8 | 18480 | 80000 | 8.43 | 41.21 |  |
| 9 | 29380 | 5000 | 23.28 | 28.29 |  |
| 10 | 14710 | 37000 | 0.14 | 19.14 |  |
| 11 | 41050 | 9000 | 6.54 | 152.73 |  |
| 12 | 13600 | 3000 | 40.22 | 200.92 |  |
| 13 | 18750 | 6000 | 18.3 | - |  |
| 14 | 33210 | 76000 | 61.89 | 13.09 |  |
| 15 | 17390 | 345000 | 24.91 | - |  |
| CPR: C reactive protein. | | | | |  |

Laboratory parameters include leukocytes, platelets, procalcitonin, and C reactive protein (CRP) in newborns infected with the pathogen.
