# Supplementary material for: Outbreak of Serratia marcescens in the Neonatal Intensive Care Unit of a Tertiary Care Hospital in Mexico
Source: Adv Med. 2023 Sep 21;2023:3281910. doi: 10.1155/2023/3281910 (PMC10539092; doi:10.1155/2023/3281910)
Supplement: Supplementary Materials — Supplementary Table 1: laboratory parameters of neonates infected with S. marcescens. Laboratory parameters such as leukocytes, platelets, procalcitonin, and C-reactive protein (CRP) in newborn infants who have been infected with the pathogen. Supplementary Table 2: screening for S. marcescens in samples collected from various areas of the Women's Hospital. Samples for bacterial culture were collected from floors and walls, air conditioners, oxygen and air intakes, furniture, medical equipment (cribs, incubators, and ventilators), water, milk, supplies, catheters, soap, computer equipment, and staff hands. [file 3281910.f1.zip › Supplementary Table 2 (1).docx]

| **Supplementary Table 2**. Samples were collected from various locations within The Women's Hospital to determine the presence of *S. marcences*. | | |
| --- | --- | --- |
| **The Women's Hospital** **areas** | **N° Cultures** | **Detection of *S. Marcences*** |
| Inhalation therapy room | 12 | Negative |
| Substitute milk bank | 18 | Negative |
| Toco-surgery | 30 | Negative |
| Expulsion rooms | 46 | Negative |
| Operating rooms | 55 | Negative |
| Central equipment and sterilization | 23 | Negative |
| Transition | 26 | Negative |
| Neonatal intensive care unit 1 | 11 | Negative |
| Neonatal intensive care unit 2 | 8 | Negative |
| Intermediate care | 10 | Negative |
| Growth and development | 18 | Negative |
| Others places | 28 | Negative |
| Cultures were taken from floors and walls, air conditioning, oxygen and air intakes, furniture, medical equipment (cribs, incubators, fans), water, milk, supplies, catheters, soap, computer equipment, and staff hands. | | |
